# Identification of Phenolic Acids and Flavonoids in Monofloral Honey from Bangladesh by High Performance Liquid Chromatography: Determination of Antioxidant Capacity

**DOI:** 10.1155/2014/737490

**Published:** 2014-06-24

**Authors:** Mohammed Moniruzzaman, Chua Yung An, Pasupuleti Visweswara Rao, Mohammad Nurul Islam Hawlader, Siti Amirah Binti Mohd Azlan, Siti Amrah Sulaiman, Siew Hua Gan

**Affiliations:** ^1^Department of Pharmacology, School of Medical Sciences, Universiti Sains Malaysia, 16150 Kubang Kerian, Kelantan, Malaysia; ^2^Human Genome Centre, School of Medical Sciences, Universiti Sains Malaysia, 16150 Kubang Kerian, Kelantan, Malaysia; ^3^Faculty of Agro Based Industry, Universiti Malaysia Kelantan, Jeli Campus, Locked Bag No. 100, 17600 Jeli, Kelantan, Malaysia; ^4^Department of Biochemistry and Molecular Biology, Faculty of Biological Sciences, Jahangirnagar University, Savar, Dhaka 1342, Bangladesh

## Abstract

The aim of the present study was to characterize the phenolic acids, flavonoids, and antioxidant properties of monofloral honey collected from five different districts in Bangladesh. A new high performance liquid chromatography (HPLC) equipped with a UV detector method was developed for the identification of the phenolic acids and flavonoids. A total of five different phenolic acids were identified, with the most abundant being caffeic acid, benzoic acid, gallic acid, followed by chlorogenic acid and trans-cinnamic acid. The flavonoids, kaempferol, and catechin were most abundant, followed by myricetin and naringenin. The mean moisture content, total sugar content, and color characteristics of the honey samples were 18.36 ± 0.95%, 67.40 ± 5.63 g/100 g, and 129.27 ± 34.66 mm Pfund, respectively. The mean total phenolic acids, total flavonoid content, and proline content were 199.20 ± 135.23, 46.73 ± 34.16, and 556.40 ± 376.86 mg/kg, respectively, while the mean FRAP values and DPPH radical scavenging activity were 327.30 ± 231.87 *μ*M Fe (II)/100 g and 36.95 ± 20.53%, respectively. Among the different types of honey, kalijira exhibited the highest phenolics and antioxidant properties. Overall, our study confirms that all the investigated honey samples are good sources of phenolic acids and flavonoids with good antioxidant properties.

## 1. Introduction

Honey is a natural product consisting of a highly concentrated solution of a complex mixture of sugar and minute quantities of other constituents, such as minerals, proteins, vitamins, organic acids, flavonoids, phenolic acids, enzymes, and volatile compounds [1, 2]. The quantity of these different compounds varies greatly depending on the floral and geographical origin of the honey. Additionally, the composition of honey is influenced by processing, handling, and storage time [3, 4].

The components in honey reported to be responsible for its antioxidant effects are flavonoids, phenolic acids, ascorbic acid, catalase, peroxidase, carotenoids, and the products of Maillard reactions [2, 4]. However, the amount and type of these antioxidants are largely dependent on the floral source or honey variety and a correlation between antioxidant activity and total phenolic content has been established [4].

Bangladesh is a country with a subtropical monsoon climate and experiences wide seasonal variations in rainfall, moderately warm temperatures, and high humidity. Honey has been used traditionally over the years by the people of Bangladesh as food and as a traditional medicine in the treatment of several diseases. Although honey is widely consumed by locals, very few data are available to support the medicinal claims of different types of honey samples from Bangladesh. Several types of honey, such as mustard (*Brassica nigra*), Kalijira (*Nigella sativa*), Padma flower* (Nelumbo nucifera),* sesame (*Sesamum indicum*), drumstick (*Moringa oleifera*), blackberry (*Syzygium cumini*), and lychee (*Litchi chinensis*), are available in Bangladesh. Although honey is widely consumed in Bangladesh, few data are available on the quality of commonly consumed honey. Investigations of honey samples collected from different floral sources and geographic locations are necessary to provide local data. Furthermore, the data available for honey reported from other countries are not applicable to Bangladesh because honey varies in antioxidant capacity, physicochemical properties, and composition based on its floral sources. In this study, we aimed to investigate different types of honey samples collected from different regions in Bangladesh and to identify the different phenolic acids, flavonoids, and antioxidant properties of some monofloral honey samples from Bangladesh. To the best of our knowledge, this is the first study to extensively investigate the different types of antioxidants present in various types of honey samples from Bangladesh.

## 2. Materials and Methods

### 2.1. Honey Samples

**Figure 1:**
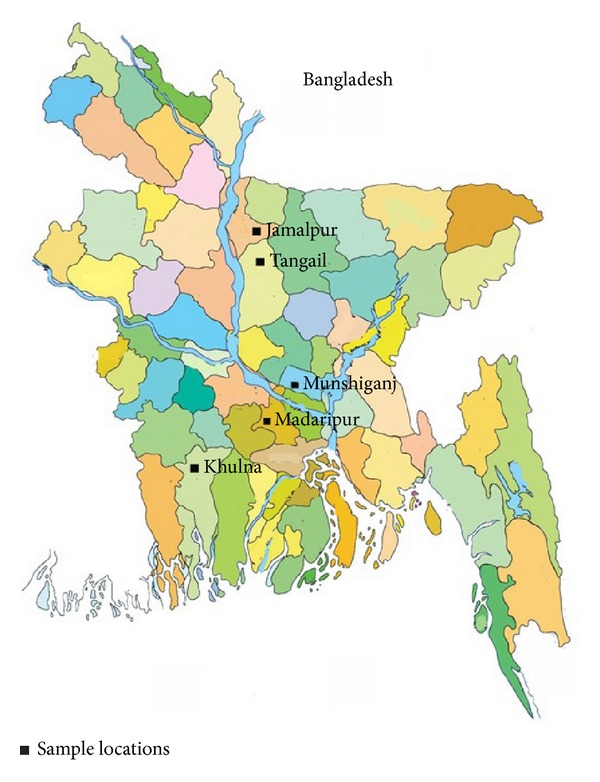
Sources of honey.

A total of ten monofloral honey (*n* = 10) samples (BH-1 to BH-10) were collected from eight different locations in five different districts (Tangail, Jamalpur, Khulna, Madaripur, and Munshigonj) in Bangladesh (Figure 1). The details of the honey, including the honey's local and scientific names, are described in Table 1. All honey collections were performed between January and December 2012. The samples were refrigerated (4-5°C) in airtight plastic containers until further analysis. All analyses were conducted in triplicate.

### 2.2. Chemicals and Reagents

The phenolic acids (gallic, syringic, caffeic, vanillic, benzoic, and trans-cinnamic acids) and flavonoids (catechin, naringenin, luteolin, hesperetin, kaempferol, apigenin, and naringin), 2,2-diphenyl-1-picrylhydrazyl (DPPH), 2,4,6-tris(1-pyridyl)-1,3,5-triazine (TPTZ), Folin-Ciocalteu's reagent, and gallic acid were purchased from Sigma-Aldrich (St. Louis, MO, USA). Sodium carbonate (Na_2_CO_3_), aluminum chloride (AlCl_3_), sodium nitrite (NaNO_2_), and sodium hydroxide (NaOH) were purchased from Merck (Darmstadt, Germany). All chemicals were of analytical grade.

### 2.3. Physical Analysis

#### 2.3.1. Moisture Content

The moisture content was determined by using a refractometric method. In general, the refractive index increases with an increase in the solid content of the sample. The refractive indices of honey samples were measured at ambient temperature using an Atago handheld refractometer (KRUSS, HRH30, Hamburg, Germany). The measurements were further corrected for the standard temperature of 20°C by adding a correction factor of 0.00023/°C. The moisture content was measured in triplicate and the percentage of moisture content that corresponds to the corrected refractive index was calculated using Wedmore's table [5].

#### 2.3.2. Total Sugar Content

Honey was suspended in Milli-Q water to make a 25% (w/v) solution. The total sugar content of each honey sample was then determined using a refractometric method (Atago handheld refractometer, ATAGO, N-1*α*, Tokyo, Japan). The percentage of sucrose content was measured per g/mL of honey.

#### 2.3.3. Honey Color Analysis

The color intensity of honey samples was measured according to the Pfund classifier. Briefly, homogeneous honey samples devoid of air bubbles were transferred into a cuvette with a 10 mm light path until the cuvette was approximately half full. The cuvette was inserted into a color photometer (HI 96785, Hanna Instrument, Cluj County, Romania). Color grades were expressed in millimeter (mm) Pfund grades when compared to an analytical-grade glycerol standard. Measurements were performed in triplicate for each sample using the approved color standards of the United States Department of Agriculture (USDA) [6].

#### 2.3.4. Color Intensity** (**ABS_450_ **)**

The mean absorbance of honey samples was determined using the method of Beretta et al. [7]. Briefly, honey samples were diluted to 50% (w/v) with warm (45–50°C) Milli-Q water and the resulting solution was filtered using a 0.45 *μ*m filter to remove large particles. The absorbance was measured at 450 and 720 nm using a spectrophotometer and the difference in absorbance was expressed as mAU.

### 2.4. Analysis of Antioxidant Properties

#### 2.4.1. Determination of Total Phenolic Compounds

The concentration of phenolic compounds in honey samples was estimated using a modified spectrophotometric Folin-Ciocalteu method [8]. Briefly, 1 mL of honey extract was mixed with 1 mL of Folin and Ciocalteu's phenol reagent. After 3 min, 1 mL of Na_2_CO_3_ (10%) solution was added to the mixture and adjusted to 10 mL with distilled water. The reaction was kept in the dark for 90 min, after which the absorbance was read at 725 nm using a T 60 UV/VIS spectrophotometer (PG Instruments Ltd., UK). Gallic acid was used to calculate a standard curve (20, 40, 60, 80, and 100 *μ*g/mL; *r* ^2^ = 0.996). The concentration of phenolic compounds was measured in triplicate. The results were reported as mean ± standard deviation and expressed as mg of gallic acid equivalents (GAEs) per kg of honey.

#### 2.4.2. Determination of Total Flavonoid Content

The total flavonoid content in each honey sample was measured using the colorimetric assay developed by Zhishen et al. [9]. Honey extract (1 mL) was mixed with 4 mL of distilled water. At the baseline, 0.3 mL of NaNO_2_ (5%, w/v) was added. After five min, 0.3 mL of AlCl_3_ (10% w/v) was added, followed by the addition of 2 mL of NaOH (1 M) 6 min later. The volume was then increased to 10 mL by the addition of 2.4 mL distilled water. The mixture was vigorously shaken to ensure adequate mixing and the absorbance was read at 510 nm. A calibration curve was created using a standard solution of catechin (20, 40, 60, 80, and 100 *μ*g/mL; *r* ^2^ = 0.998). The results were expressed as mg catechin equivalents (CEQ) per kg of honey.

#### 2.4.3. Ferric Reducing/Antioxidant Power Assay (FRAP Assay)

The FRAP assay was performed according to a modified method described by Benzie and Strain [10]. Briefly, 200 *μ*L of properly diluted honey (0.1 g/mL) was mixed with 1.5 mL of FRAP reagent. The reaction mixture was then incubated at 37°C for 4 min and its absorbance was read at 593 nm against a blank that was prepared with distilled water. Fresh FRAP reagent was prepared by mixing 10 volumes of 300 mM/L acetate buffer (pH 3.6) with 1 volume of 10 mM TPTZ solution in 40 mM HCl containing 1 volume of 20 mM ferric chloride (FeCl_3_ *·*6H_2_O). The resulting mixture was then prewarmed at 37°C. A calibration curve was prepared using an aqueous solution of ferrous sulfate (FeSO_4_·7H_2_O) at 100, 200, 400, 600, and 1000 *μ*M. FRAP values were expressed as micromoles of ferrous equivalent (*μ*M Fe [II]) per kg of honey.

#### 2.4.4. DPPH Free Radical-Scavenging Activity

The antioxidant properties of each honey sample were also investigated by determining the free radical-scavenging activity of the DPPH radical based on the method proposed by Ferreira et  al. [11]. Briefly, honey extract (0.5 mL) was mixed with 2.7 mL of methanolic solution containing DPPH radicals (0.024 mg/mL). The mixture was vigorously shaken and left to stand for 15 min in the dark (until the absorbance stabilized). The reduction of the DPPH radical was determined by measuring the absorbance of the mixture at 517 nm.

Butylated hydroxytoluene (BHT) was used as a reference. The radical-scavenging activity (RSA) was calculated as the percentage of DPPH discoloration using the following equation: %  RSA = ([*A*_DPPH_ − *A*_*S*_]/*A*_DPPH_) × 100, where *A*_*S*_ is the absorbance of the solution when the sample extract is added at a particular level and *A*_DPPH_ is the absorbance of the DPPH solution.

#### 2.4.5. Proline Content

The proline content in the honey samples was measured using a method established by the International Honey Commission (IHC) [12]. Briefly, approximately 5 g of honey was transferred to a beaker and dissolved in 50 mL water. The solution was quantitatively transferred to a 100 mL volumetric flask before further dilution to 100 mL with distilled water. After that, approximately 0.5 mL of the sample solution was transferred to a tube, while 0.5 mL of water (blank test) was transferred to a second tube and 0.5 mL of proline standard solution was dispensed into three other tubes. To each tube, approximately 1 mL of formic acid and 1 mL of ninhydrin solution were added. The tubes were capped carefully and shaken vigorously for 15 min. The tubes were then placed in a boiling water bath for 15 min and immersed below the level of the solution. The tubes were further transferred to another water bath and incubated at 70°C for 10 min. Approximately 5 mL of the 2-propanol water solution was added to each tube followed by immediate capping. The tubes were left to cool for approximately 45 min after removal from the 70°C water bath and the absorbance values were measured at 510 nm (near the maximum wavelength).

### 2.5. HPLC Analysis of Phenolic Acids and Flavonoids 

#### 2.5.1. Extraction of Phenolic Compounds

A modified solid-phase extraction (SPE) procedure was developed to extract the phenolic compounds present in honey. Briefly, honey (2 g) was dissolved in 10 mL of acidified deionized water (pH 2) that was pH adjusted using orthophosphoric acid (85%) and the solution was run through Bond Elut C18 cartridges (3 mL × 500 mg) (Agilent Technologies, Santa Clara, CA, USA). The cartridges were preconditioned by sequentially passing 3 mL of methanol followed by the addition of acidified water (pH 2) dropwise. An aqueous honey solution (5 mL) was then applied to the preconditioned cartridges drop by drop to ensure efficient adsorption of the investigated compounds to the bonded phase. The adsorbed phenolic compounds were then eluted from the cartridges with 2 mL of 90% (v/v) methanol/water solution dropwise. The eluates were collected and evaporated to dryness under a gentle flow of nitrogen gas. Finally, the extract was reconstituted with 1 mL of methanol before HPLC analysis.

#### 2.5.2. HPLC Analysis

A new HPLC method was developed for the detection of phenolic acids and flavonoids by employing an HPLC system (Waters 2695, Milford, MA, USA) equipped with a photodiode array detector (Waters 2996). The HPLC column was a Merck Purospher Star, RP-18e (150 × 4.6 mm, 5 *μ*m), fitted with a guard cartridge that had been packed with a similar type of stationary phase (Merck). A linear gradient flow was employed at a flow rate of 0.5 mL/min throughout and the total analytical time was approximately 40 min.

The binary mobile phase consisted of solvent A (ultrapure water with 0.1% phosphoric acid) and solvent B (pure methanol with 0.1% phosphoric acid). Elution from the column was achieved with the following gradients: 0 to 20 min of solvent B, increasing from 10% to 85%; 20 to 25 min of solvent B at 85% throughout; 25 to 26 min of solvent B from 85% decreasing to 10%; and a final composition at 10% that was kept constant to 40 min. The detection wavelength was set between 200 and 450 nm, with specific monitoring conducted at 220 nm. Identification of the phenolic and flavonoid compounds was performed by comparing the retention times of the analytes with reference standards.

### 2.6. Statistical Analysis

Assays were performed in triplicate and the results were expressed as the mean values with standard deviations (SD). The significant differences represented by letters were obtained by a one-way analysis of variance (ANOVA) followed by Tukey's honestly significant difference (HSD) post hoc test (*P* < 0.05). Correlations were established using Pearson's correlation coefficient (*r*) in bivariate linear correlations (*P* < 0.01). These correlations were calculated using Microsoft office Excel 2007 and SPSS version 16.0 (IBM corporation, New York, USA).

## 3. Results and Discussion

### 3.1. Moisture Content

**Figure 2:**
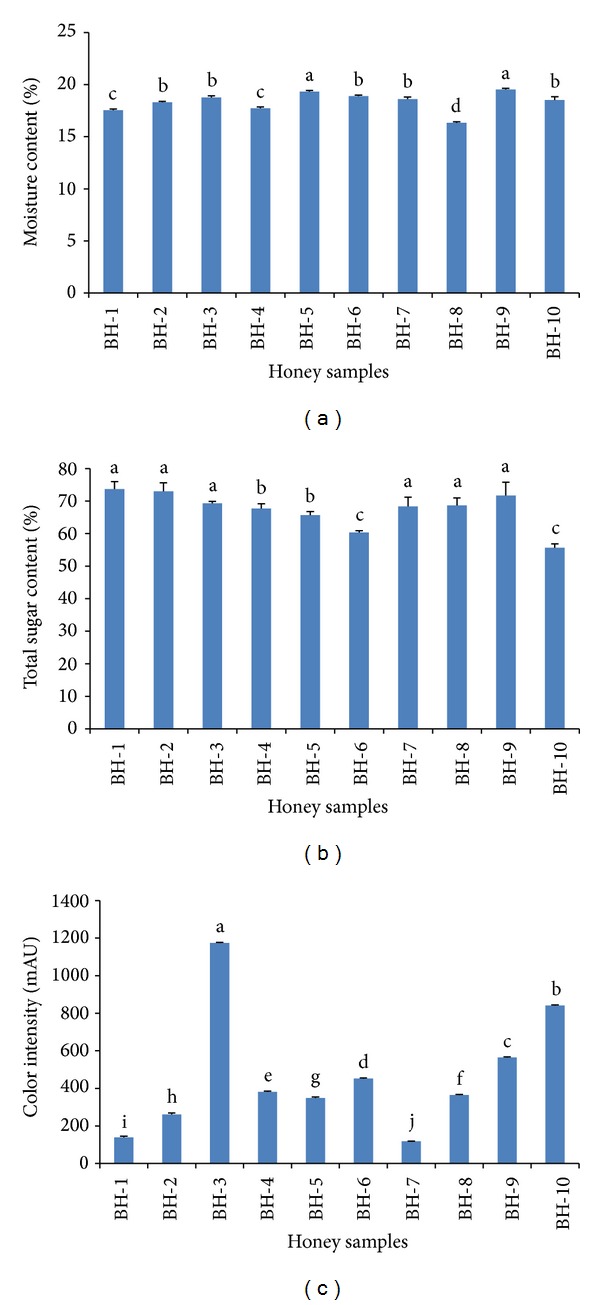
Physical parameters such as (a) moisture content, (b) total sugar content, and (c) color intensity of the investigated honey samples.

Moisture is a physicochemical parameter of honey associated with the climatic conditions of the location from which the honey was gathered as well as the degree of honey maturity [13]. It is one of the key factors that determine the quality of honey. In the present investigation, the moisture content was between 16.33 and 19.53% (Figure 2(a)), which was within the limit of ≤20% set by the international regulations for honey quality [14]. The moisture content present in honey samples is important because it contributes to the honey's ability to resist fermentation and crystallization during storage [15, 16].

Significant variations in moisture content were observed in the investigated monofloral honey. The mean moisture content was 18.36 ± 0.95%, with the lowest moisture content (16.33%) exhibited by sample BH-8, which was collected in June 2012, while sample BH-9 exhibited the highest moisture content (19.53%). Low moisture content in honey can confer a protective effect against microbial attack, especially during long term storage [15, 16]. On the other hand, high moisture content can promote honey fermentation during storage as a result of the activity of osmotolerant yeasts and the consequent formation of ethyl alcohol and carbon dioxide [16]. The lower moisture content exhibited by most of the investigated honey samples ensures the better quality of these honey samples, which allows them to be stored for a longer duration.

The moisture contents of the analyzed samples were consistent with previously reported values of honey samples from Bangladesh of 12.79% to 22.32% [17]. Moreover, the moisture contents for the investigated honey samples were similar to those from countries of similar climates, including honey from India (17.20 to 21.60%) [16] and Malaysia (14.86–17.53%) [18].

### 3.2. Total Sugar Content

The total sugar content of the investigated honey samples ranged from 55.67 to 73.67%, with a mean value of 67.40 ± 5.63% (Figure 2(b)). According to the European Commission directive, the total glucose and fructose content of honey should exceed 60 g per 100 g of honey for natural honey [19]. All of the investigated honey samples contained total sugar contents higher than the recommended level, except for sample BH-10 (55.67%). The lower total sugar content of sample BH-10 can be attributed to the processing or storage and cannot be attributed to the honey source because sample BH-1, which contained the highest total sugar content (73.67%), was from a similar source.

Sugars generally constitute the main components of honey, regardless of type, with reducing sugars (mainly fructose and glucose) making up the majority. In a previous report, the total sugar content of Bangladeshi honey samples was low, ranging from 42.80 to 60.67% [17]. Although the honey came from the same country, the variation observed could have been due to the different floral sources. However, the sugar content of the investigated honey samples is reported to be generally lower than that reported for honey from Algeria (62.80 to 70.00%), which tends to be sweeter [20].

### 3.3. Color Characteristics

**Figure 3:**
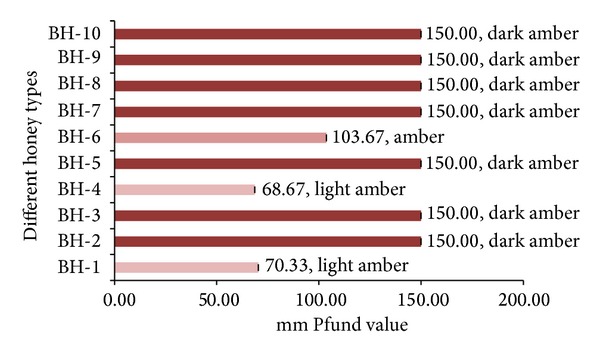
Color characteristics of monofloral honey from Bangladesh.

Honey color based on the USDA-approved color standards [6] is one of the primary characteristics of honey classification. Honey color differs naturally, ranging from light yellow to amber, dark amber, and black (in extreme cases) and sometimes green or red hues may even occur [18]. The color of untreated honey depends on its botanical origins. For this reason, color is very important for the classification of monofloral honey for commercial use. The higher the Pfund value is, the darker the honey color should be. In the present study, the honey color ranged from 70.33 to 150.00 mm Pfund (Figure 3). The majority of the investigated honey samples were dark amber in color, with a mean Pfund value of 150.00, indicating their better qualities, as honey color is reported to be dependent not only on various components, such as ash and potential alkalinity, but also on the concentrations of antioxidants, such as phenolic acids and flavonoids. Our results are similar to those reported for some honey samples from Malaysia (38.33–150.00) [18] and Algeria (107.00–150.00) [20].

### 3.4. Color Intensity

**Figure 4:**
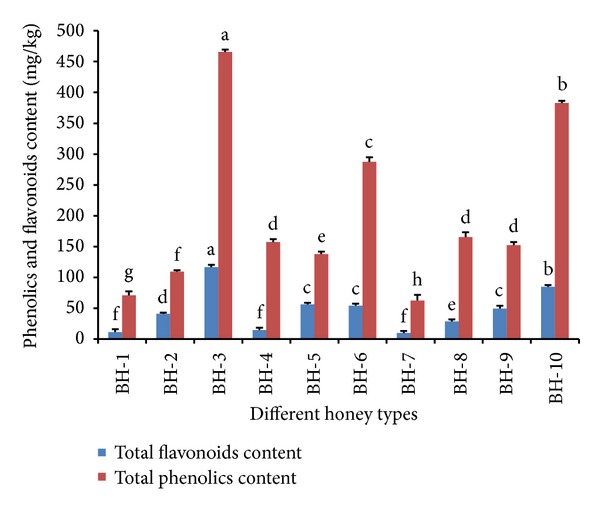
Total phenolic and flavonoid contents of the monofloral honey from Bangladesh.

The color intensity of honey is represented by the ABS_450_, which also indicates the presence of pigments such as carotenoids and flavonoids, which are also known to contribute to antioxidant properties [18, 20]. In the present study, the honey's ABS_450_ values ranged between 117 and 1174 mAU (Figure 2(c)). Sample BH-3, which was among the highest Pfund values, also showed correspondingly the highest color intensity (1174.00 ± 3.61 mAU), indicating its better antioxidant properties. High color intensity was also observed in two other honey samples (BH-10 and BH-9 at 841.67 and 564.33 mAU, resp.), which correspondingly had high antioxidant potential (Figure 4),as also reported by [18, 21, 22].

Comparatively, the ABS_450_ values were reported to be between 724 and 1188 mAU in honey samples from Algeria [20], between 25 and 3413 mAU in honey samples from Italy [7], between 70 and 495 mAU in honey samples from Slovenia [3], between 254 and 2034 mAU in honey samples from Bangladesh [17], and between 524 and 1678 mAU in honey samples from India [16]. The comparatively high ABS_450_ values in the investigated honey samples indicate their high antioxidant properties and purity.

### 3.5. Total Phenolic Content

The concentration and type of phenolic substances depend on the floral origin of the honey and are mainly responsible for its biological activities [1]. The total phenolic content (mg/kg of honey) was found to vary significantly among the various honey samples from Bangladesh (Figure 4), which may be due to their different botanical and regional origins. The mean total phenolic content of the studied honey samples was 199.20 mg/kg, with the highest phenolic content recorded by sample BH-3 at 465.68 ± 3.75 mg/kg, indicating its superior antioxidant potential. kalijira honey (BH-3) which is only available in the winter season in Bangladesh is significantly different from the samples analysed. Two previous studies [22, 23] reported that black cumin (*Nigella sativa*) is a good source of phenolic compounds and exhibits high antioxidant capacity. It is plausible that the high phenolic content in the plant parts of the black cumin (*Nigella sativa*) tree is transferred into the nectar collected by the bees and introduced to the honey, contributing to the high phenolic contents observed in this type of honey.

The mean phenolic content of the investigated honey samples is higher than that of lime honey (83.7 mg/kg) [3], acacia honey from Slovenia (44.8 mg/kg) [3], and honey samples from Burkina Faso (74.38 mg/kg) [24], indicating its superior antioxidant properties. In a previous study, the phenolic contents of honey samples from Bangladesh that were stored for more than one year were slightly higher (152.4 to 688.5 mg/kg) [17]. It has been reported that the botanical [3, 7] and geographical region from which the honey is collected not only affects its phenolic and flavonoid concentrations but also affects its pollen distribution and, ultimately, its antioxidant activities.

### 3.6. Total Flavonoid Content

Flavonoids are low molecular weight phenolic compounds responsible for the aroma and the antioxidant potential of honey. The total flavonoid content in the investigated honey samples ranged from 11.46 to 116.67 mg_catechin_/kg (Figure 4). As with the phenolic content, the highest flavonoid concentration was also shown by sample BH-3. The results are in agreement with those of several previous studies, in which it was found that honey samples with higher polyphenol content will also yield high flavonoid levels [18, 20]. On average, the mean flavonoid content (46.73 mg/kg) of the investigated honey samples from Bangladesh was higher than that of honey samples from Slovenia (20.57 mg/kg) [24], indicating the superior antioxidant properties of these honey samples.

### 3.7. Determination of Total Antioxidant Content by FRAP Assay

**Figure 5:**
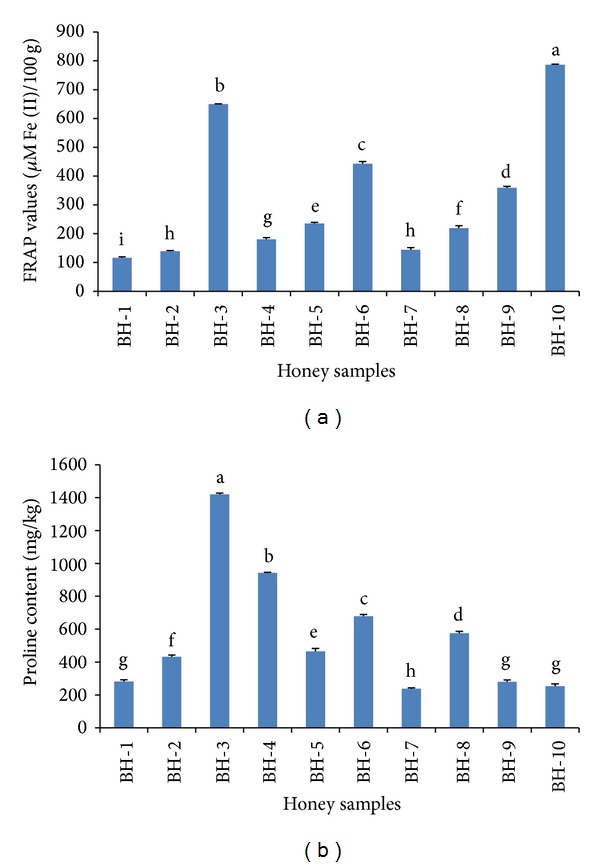
(a) FRAP values and (b) proline content of monofloral honey from Bangladesh.

The FRAP assay is used to measure the total antioxidant content of honey. It directly estimates the presence of either antioxidants or reductants, depending on the ability of the analyte to reduce the Fe^3+^/Fe^2+^ redox couple [7]. The FRAP values for the investigated honey samples from Bangladesh ranged from 116.00 to 786.22 *μ*M Fe (II)/100 g of honey. There were significant differences among the FRAP values for the different types of honey (Figure 5(a)), suggesting that they have different antioxidant potentials. Samples BH-3 and BH-10 again contained the highest FRAP values (786.22 *μ*M Fe (II)/100 g of honey) compared to the other samples. The mean FRAP value (327.30 *μ*M Fe (II)/100 g) of the investigated honey samples from Bangladesh is higher than that of acacia honey from Burkina Faso (79.5 *μ*M Fe (II)) and chestnut (388.6 *μ*M Fe (II)) and chicory honey (209.5 *μ*M Fe (II)) [7] from Slovenia, acacia honey [71.00 *μ*M Fe (II)] and lime honey [118.8 *μ*M Fe (II)] [3], suggesting the high quality of the honey samples from Bangladesh, as indicated by their high antioxidant potentials.

### 3.8. Proline Content

Proline is one of the most abundant amino acids in honey and is therefore usually selected as the standard for quantifying the amino acid content. Moreover, the proline content is an indicator for honey and can also reveal adulteration, as indicated by a proline level of less than 183 mg/kg [24]. Proline content in honey is a criterion of honey ripeness and robustness [12]. In our study, all of the investigated honey samples contained high amounts of proline, which ranged from 237.51 to 1419.33 mg/kg (Figure 5(b)). Similar to the phenolic acid and flavonoid contents, honey sample BH-3 again contained the highest proline content (1419 mg/kg), indicating its superior antioxidant potential.

Generally, the proline contents of the investigated samples were higher than those previously reported from Bangladesh (106–681 mg/kg) [17], India (133–674 mg/kg) [16], and Malaysia 392.85 mg/kg [18].

### 3.9. DPPH Free Radical-Scavenging Activity

**Figure 6:**
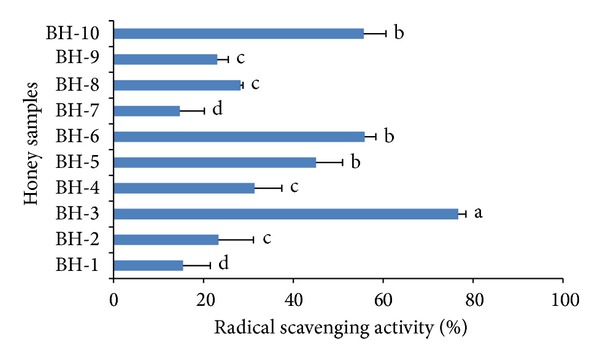
DPPH radical-scavenging activities of the monofloral honey from Bangladesh.

In evaluating the radical-scavenging potential of a sample including honey, the DPPH assay is frequently used. Usually, a high DPPH scavenging activity reflects high levels of antioxidant potential. The mean DPPH radical-scavenging activity of the investigated honey samples was 36.95%. Sample BH-3 again exhibited the highest DPPH radical-scavenging activity (76.68%), which could be attributed to its higher phenolic acid and flavonoid content (Figure 6), as it has been reported that the antioxidant potential of honey is directly proportional to the amount of phenolic acids and flavonoids present [7]. Overall, the DPPH scavenging and antioxidant potentials of the honey samples from Bangladesh were higher than that previously reported for some Malaysian honey samples [18], Indian honey samples [16], and Algerian honey samples [20], again indicating their good quality.

### 3.10. Identification and Determination of Phenolic Compounds by HPLC

**Figure 7:**
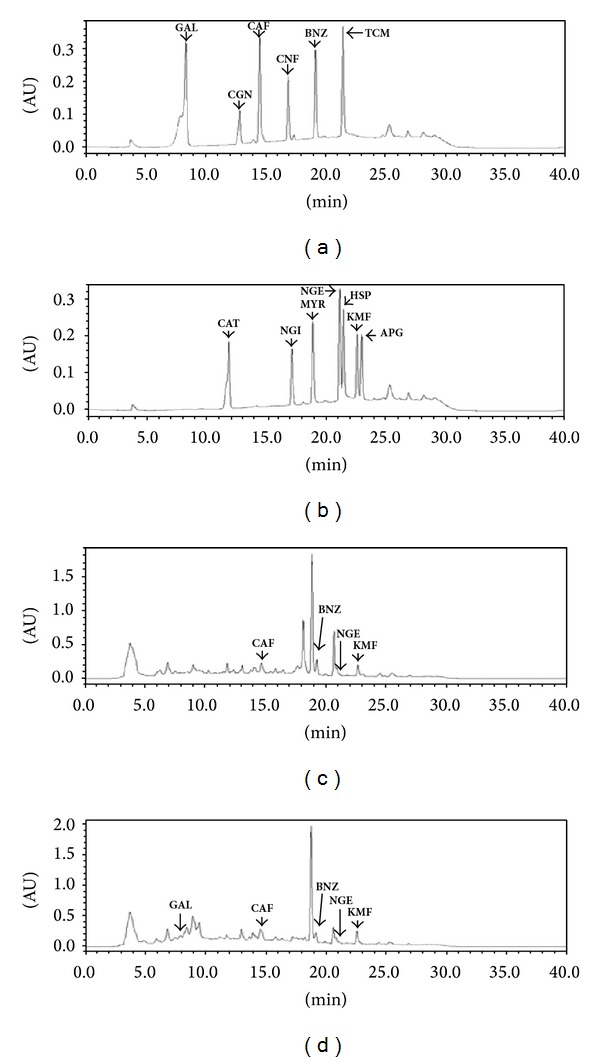
Typical chromatograms for (a) phenolic acid standards, (b) flavonoids standards, (c) phenolic acids and flavonoids in honey sample BH-3, and (d) phenolic acids and flavonoids in honey sample BH-6. GAL: gallic acid, CGN: chlorogenic acid, CAF: caffeic acid, CNF: coniferic acid, BNZ: benzoic acid, TCM: trans-cinnamic acid, CAT: catechin, NGI: naringenin, MYR: myricetin, NGE: naringenin, HSP: hesperitin, KMF: kaempferol, and APG: apigenin.

**Figure 8:**
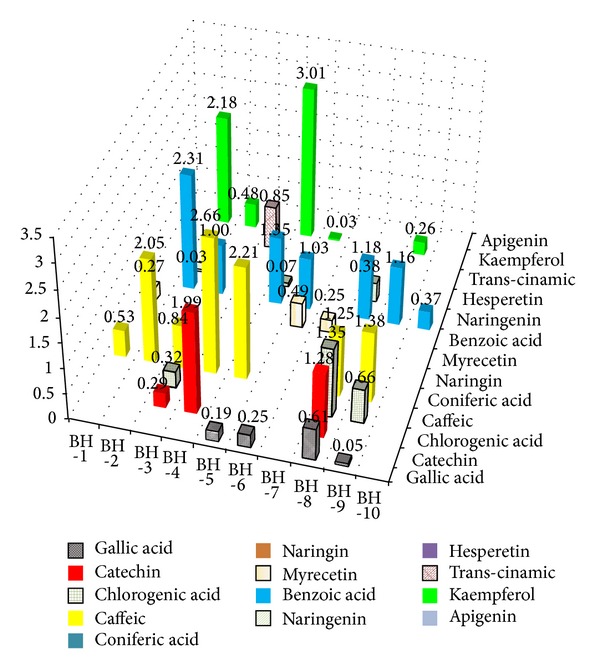
Phenolic acids and flavonoid compounds detected in the different types of honey samples from Bangladesh using HPLC.

A modified solid-phase extraction (SPE) sample preparation method was developed for the extraction of phenolic compounds in honey based on the method published by Khalil et al. [2]. A total of thirteen different phenolic compounds consisting of seven flavonoids (catechin, naringin, myricetin, naringenin, hesperetin, kaempferol, and apigenin) and six phenolic acids (gallic acid, chlorogenic acid, caffeic acid, coniferic acid, benzoic acid, and trans-cinnamic acid) were investigated (Figures 7(a), 7(b), and 8). From this number, five phenolic acids (gallic acid, chlorogenic acid, caffeic acid, benzoic acid, and trans-cinnamic acid) and four flavonoids (catechin, myricetin, naringenin, and kaempferol) were detected in the honey samples from Bangladesh. The differences in the presence of the phenolic compounds in the investigated monofloral honey from Bangladesh may be due to their diverse botanical and regional sources.

Among the samples that contained between five and six phenolic acids were samples BH-3, BH-5, BH-8, and BH-9, corresponding with their high color intensity, total phenolic acids, total flavonoids, FRAP assay, and DPPH scavenging activities, as previously shown. However, based on the findings by Aljadi and Yusoff [25], the antioxidant potential of honey as well as of propolis is mainly contributed by phenolic acids, as was also shown in our correlation analysis in the later part of the study.

Caffeic acid and benzoic acid were the most abundant phenolic compounds (70%) among all the phenolic acids and were detected in seven of the samples. This was followed by kaempferol (detected in five honey samples) and gallic acid (detected in four honey samples). Catechin, chlorogenic acid, myricetin, and naringenin were each detected in three of the honey samples, while apigenin was detected in a single sample. Coniferic acid, naringenin, and hesperetin were not present in any of the investigated honey samples. In a previous study, hesperetin and apigenin were detected in honey samples from Portugal [26], while apigenin was identified previously in a type of honey from Malaysia named* gelam honey* [2].

In addition to apigenin, honey samples from Malaysia have also been reported to contain catechin, gallic acid, caffeic acid, syringic acid, benzoic acid, naringenin, trans-cinnamic acid, and kaempferol apigenin [2]. In another study,* gelam* and coconut honey samples from Malaysia were found to contain gallic, caffeic, benzoic, ferulic, and cinnamic acids [25]. Honey samples from Australia contained gallic acid, caffeic acid, chlorogenic acid, myricetin, kaempferol, coumaric acid, ferulic acid, and quercetin [27]. Honey samples from Portugal contained coumaric acid, ferulic acid, quercetin, vanillic acid, rosmarinic acid, and kaempferol [26, 28]. The variations observed may be due to the different botanical sources of honey from Bangladesh compared with honey from other parts of the world.

Kaempferol, gallic acid, and catechin are well known for their antioxidant properties, as described in previous studies [25, 28]. The phenolic compounds present in the investigated honey samples from Bangladesh possess several medicinal properties, such as antioxidant, antibacterial, and antimicrobial properties. In particular, benzoic acid, chlorogenic acid, and caffeic acid were found to exhibit antibacterial properties [25, 28].

The most abundant phenolic acids were caffeic acid (0.00–2.66 mg/kg), benzoic acid (0.00–2.31 mg/kg), gallic acid (0.00–0.61 mg/kg) and chlorogenic acid (0.00–1.35 mg/kg), followed by trans-cinnamic acid (0.00–0.85 mg/kg). The gallic acid contents in the investigated Bangladeshi honey are similar to those of honey samples reported from Malaysia (0.43 mg/kg) [2] but lower than those of Australian honey (1.58 mg/100 g) [27], while chlorogenic acid was detected in Polish honey at 0.098 to 3.342 mg/100 g [29]. Similar to our findings, trans-cinnamic acid was reported at 0.01 to 0.50 mg/kg in Malaysian honey samples [2]. Benzoic acid was found at 0.20–11.33 mg/kg in different honey samples from Malaysia [2]. On the other hand, caffeic acid was found at 0.001 mg/kg in Malaysian Borneo tropical honey [2], 1.08 mg/100 g in Australian honey [27], and 0.021–0.101 mg/100 g in Polish honey [29].

For flavonoids, kaempferol (0.00–3.01 mg/kg) and catechin (0.00–1.99 mg/kg) were most abundant, followed by myricetin (0.00–0.49 mg/kg) and naringenin (0.00–0.38 mg/kg). Kaempferol was detected at 0.02 to 0.81 mg/kg in Malaysian honey [2] and 0.01 to 0.099 mg/100 g in Polish honey [29].

The majority of phenolic compounds are small molecular weight compounds that have the tendency to elute faster from the column, with retention times varying between 8 and 24 min. When some of the compounds coeluted during the analysis, gradient elution was employed to produce better separated peaks and facilitate identification. In addition, there were some unknown peaks having similar spectra to those of flavonoids and phenolic acids. Unfortunately, they could not be fully identified due to the lack of standards for HPLC for these compounds.

Previous reports on phenolics compound concentrations in honey that utilized spectrophotometry methods [2, 30] typically reported higher levels of phenolic compounds compared with those determined by HPLC methods. This could be due to the interference of nonphenolic materials present in the extracts, which may interfere with spectrophotometric analysis by causing higher readings, as suggested by Escarpa and González [30]. Overall, the phenolic compounds detected in the honey samples from Bangladesh have high antioxidant properties and may show promising pharmacological effects in the treatment of chronic diseases, which should be further confirmed in clinical trials.

### 3.11. Correlation Analysis

Several strong correlations were observed amongst some of the biochemical and antioxidant parameters. A strong correlation was established between the color intensities of the honey samples and their antioxidant parameters: phenolic acids, flavonoids, DPPH, and FRAP values at 0.943, 0.926, 0.838, and 0.894. The color intensity of the honey also increased with higher phenolic and flavonoid content in the honey. For example, BH-3 honey, which had the highest color intensity, also showed the highest phenolic content, suggesting that honey color pigments conferred by the phenolic compounds may play a role in the observed antioxidant activities of honey samples.

Another strong correlation was established between the ABS_450_, DPPH, and FRAP values, again indicating the involvement of pigments that ultimately confer antioxidant potential to honey. In a previous study conducted by Bertoncelj et al. [3], a strong correlation (*r* = 0.850) between the ABS_450_ and FRAP values was established in Slovenian honey. The correlation between the ABS_450_ and FRAP values was also high (*r* = 0.83) in Indian honey [16], indicating that ABS_450_, DPPH, and FRAP values are good predictors of antioxidant properties in honey. Thus, the higher correlations established in our study (ABS_450_ and DPPH, *r* = 0.838; ABS_450_ and FRAP, 0.894) suggest that the investigated monofloral honey samples from Bangladesh have a stronger antioxidant capacity when compared to Indian and Slovenian honey.

A positive significant linear correlation was also observed for the phenolic and flavonoid contents with the DPPH (*r* = 0.915) and FRAP values (*r* = 0.876), respectively, which indicates the involvement of these compounds with the antioxidant properties of the investigated honey samples from Bangladesh, as phenolic acids and flavonoids are well known for their antioxidant potential. Generally, these strong positive correlations suggest that the monofloral honey samples from Bangladesh have strong antioxidant potential.

## 4. Conclusion

This is the first study to identify the phenolic compounds in honey samples from Bangladesh. Caffeic acid and benzoic acid were the most abundant phenolic compounds (70%) among all the phenolic acids, followed by kaempferol and gallic acid. In addition to these compounds, catechin, chlorogenic acid, myricetin, naringenin, and apigenin were also detected. Coniferic acid, naringenin, and hesperetin were not present in any of the investigated honey samples. The mean total phenolic acids, total flavonoid content, and proline content were 199.20, 46.73, and 556.40 mg/kg, respectively, while the mean FRAP values and DPPH radical-scavenging activity were 327.30 *μ*M Fe (II)/100g and 36.95%, respectively. Among the different types of honey, the honey samples collected from the* Nigella sativa* plant typically exhibited the highest phenolic content and antioxidant properties. A strong correlation was established between the color intensities of the honey samples and their antioxidant parameters: phenolic acids, flavonoids, and DPPH and FRAP values. However, further studies investigating on several different types of honey samples including Kalijira, Padma, and Teel honey are warranted. Overall, our study confirms that all the investigated honey samples from Bangladesh are good sources of phenolic acids and flavonoids, which confer their good antioxidant potential.

## Figures and Tables

**Table 1:** Floral type and source of the investigated Bangladeshi monofloral honey.

Sample ID number	Local and scientific name of source	Location of source	Time of collection
BH-1	Mustard flower *(Brassica nigra) *	Mirzapur, Tangail	December 2012
BH-2	Mustard flower *(Brassica nigra) *	Melandah, Jamalpur	February 2012
BH-3	Kalijira *(Nigella sativa) *	Khulna Sadar, Khulna	March 2012
BH-4	Padma flower *(Nelumbo nucifera) *	Khalishpur, Khulna	March 2012
BH-5	Mustard flower *(Brassica nigra) *	Shakipur, Tangail	December 2012
BH-6	Mustard flower *(Brassica nigra) *	Shakipur, Tangail	November 2012
BH-7	Mustard flower *(Brassica nigra) *	Shakipur, Tangail	December 2012
BH-8	Teel/sesame *(Sesamum indicum) *	Kalkini, Madaripur	June 2012
BH-9	Mustard flower *(Brassica nigra) *	Tangail Sadar, Tangail	January 2012
BH-10	Mustard flower *(Brassica nigra) *	Gazaria, Munshigonj	May 2012
